# Development and experimental validation of dephosphorylation-related biomarkers to assess prognosis and immunotherapeutic response in gliomas

**DOI:** 10.3389/fimmu.2024.1488894

**Published:** 2025-01-03

**Authors:** Hui Tang, Xuping Yang, Guoqian Li, Ke Peng, Yang Sun, Longyang Jiang, Yilan Huang

**Affiliations:** ^1^ Department of Pharmacy, The Affiliated Hospital of Southwest Medical University, Luzhou, China; ^2^ School of Pharmacy, Southwest Medical University, Luzhou, China; ^3^ Department of Pharmacy, Chengdu Chengnan Jinhua Hospital, Chengdu, China; ^4^ Department of Pharmacy, Renshou County Traditional Chinese Medicine Hospital, Meshan, China; ^5^ Department of Pharmaceutical Analysis, Central Nervous System Drug Key Laboratory of Sichuan Province, Luzhou, China

**Keywords:** dephosphorylation-related genes, glioma, biomarkers, prognosis, temozolomide, chemotherapy drug sensitivity, drug resistance

## Abstract

**Background:**

Gliomas are common aggressive brain tumors with poor prognosis. Dephosphorylation-related biomarkers are in a void in gliomas. This study aims to construct a validated prognostic risk model for dephosphorylation, which will provide new directions for clinical treatment, prognostic assessment, and temozolomide (TMZ) resistance in glioma patients.

**Methods:**

Screening dephosphorylation-related genes (DRGs) and transcriptome expression data from The Cancer Genome Atlas (TCGA), Molecular signatures database (MSigDB) and constructing risk scoring models. Kaplan-Meier (K-M), nomogram and ROC curve were used to assess the predictive efficacy of the model. Gene set enrichment analysis (GSEA), immune cell infiltration, immunotherapy response and chemotherapeutic drug sensitivity analysis were performed in this study. The correlation between chemotherapeutic drugs and the half maximal inhibitory concentration (IC_50_) values of 12 DRGs was analyzed. Cell division cycle 25A (CDC25A) and TMZ were screened and verified by experiments. Quantitative Real-Time PCR (qRT-PCR) detection of mRNA expression of 12 genes in human normal glial cells and two glioma cell lines. Transfection techniques overexpressed and knocked down CDC25A. qRT-PCR and Western Blot (WB) were used to detect the mRNA and protein expression levels of CDC25A. Subsequently, verify the effect of CDC25A on TMZ resistance in glioma cells.

**Results:**

The model established in this study was able to accurately predict the prognosis of glioma patients. Besides, there were significant differences in GSEA, immune cell infiltration, immunotherapeutic response and chemotherapeutic drug sensitivity analysis between glioma patients in the high and low risk groups. The results of CCK8 experiments showed that overexpression of CDC25A increased the susceptibility of U251 and LN229 cells to TMZ, and knockdown of CDC25A attenuated the susceptibility of U251 and LN229 cells to TMZ.

## 1 Introduction

According to the latest global cancer statistics report, there are nearly 9.7 million deaths from cancer worldwide, including about 320,000 new cases of brain and nervous system tumors, and 250,000 deaths (1). It is estimated that about 600,000 people in the United States will die of cancer by 2024. The mean annual adjusted incidence of central nervous system tumors was 25.34/100,000, and the 5-year relative survival rate after diagnosis of malignant brain tumors or other central nervous system tumors was 35.7% (2). At present, the main treatment methods of tumor include surgical resection, radiotherapy, chemotherapy, immunotherapy and targeted therapy (3). Glioma is a common malignant brain tumor. Due to the invasive growth characteristics of gliomas, low sensitivity to radiation, chemotherapy, and mostly late stage diagnosis (4–8). It is difficult to achieve complete curative resection, and the survival rate of patients is still limited. Further research and exploration of more effective treatment methods are needed to improve the survival rate of patients.

Phosphorylation and dephosphorylation are classical reversible post-translational modifications of proteins in eukaryotes, which are mainly catalyzed by protein kinases and phosphatases (9). Nearly one third of biological functions in cells are regulated and controlled by protein flow acidification, such as cell division, cell growth and development, proliferation and regulations (10–14). Currently, phosphorylation and dephosphorylation of many signaling pathways, including tyrosine kinases, calreticulin-linker complexes, etc., and dysregulation of their phosphorylation/dephosphorylation cascades has been shown to manifest in various types of cancers (10, 13, 15–19).

In recent years, with the deepening of molecular pathology, biomarkers have played a pivotal role in the diagnosis and treatment of glioma (20–22). The research of new biomarkers for glioma is gradually increasing, such as multicellular biomarkers of drug resistance, biomarkers of voltage-gated sodium channel β 3 subunit, cyclin dependent kinase 2, Insulin-like growth factor-binding proteins, MUC16 mutation (23–28). Studies on biomarkers related to protein post-translational modification, including glycosylation, ubiquitination and palmitoylation, to predict the prognosis of glioma patients have gradually increased. However, studies on biomarkers related to phosphorylation and dephosphorylation in glioma are still blank. Our study is the first paper to investigate biomarkers related to glioma and dephosphorylation, and we hope that our study will fill this research gap and inspire others.

Therefore, we started from dephosphorylation, searches for dephosphorylation related biomarkers and constructs a dephosphorylation-related genes (DRGs) model to predict the survival and prognosis of glioma patients, explored potential molecular mechanisms and hope to inspire others. The strength of this study is to fill the gap of DRGs in glioma biomarkers. In addition, we analyzed the differences in immune cell infiltration, immunotherapy and chemotherapeutic drug sensitivity in glioma patients in high and low risk groups. Finally, it is worth mentioning that this study was conducted to experimentally validate the effect of gene CDC25A on temozolomide (TMZ) resistance, which is hoped to provide a reference for the individualized treatment of temozolomide-resistant glioma patients in the clinic.

## 2 Methods

### 2.1 Data extractions

Transcriptome expression data of 511 glioma patients and clinical survival data of 515 glioma patients were downloaded from TCGA database and the data profiles were extracted and normalized (FRKM to TPM) using R software. 9 of the 515 glioma patients did not have transcriptome expression data, and after elimination, we ended up with 506 glioma patients who had both transcriptome expression data information and survival data. Screening of DRGs from the Molecular Signatures Database (MSigDB) database (GOBP_Dephosphorylation.v.7.5.1), and 8 genes not included in the TCGA database were removed, which was finally collated into 417 dephosphorylation-related candidate gene set.

### 2.2 Construction and verification of risk score modle

Least Absolute Shrinkage and Selection Operator (LASSO) regression analysis compresses the variable regression coefficient in the regression model by generating a penalty function lambda value (λ) to prevent over-fitting and solve the problem of multicollinearity. In our study, 417 DRGs were included in LASSO regression. With the increase of λ, the regression coefficient β of 417 DRGs decreased, and some of them would become 0, indicating that the DRGs contributed little to the model at this time and could be eliminated. The “Glmnet” package in R software was utilized for cross-validation to determine the optimal λ value. The results indicated that there were two penalty values. One was the λ value when the mean square error was at its minimum, namely λ.min. The other was the λ value of the simplest model obtained within a variance range of λ.min, namely λ.1se. In this study, when λ was screened out as 17, the model fitting effect was the highest. Next, a multifactor Cox regression analysis was performed on the results of the LASSO regression analysis, from which 12 genes associated with dephosphorylation with prognostic significance were identified, and a prognostic risk model was constructed accordingly. The risk score was calculated by multiplying the multivariate Cox proportional hazards regression coefficients by their gene expression levels for each patient. The formula for risk score:

$\text{Risk score = }\sum_{i}^{n}\;Coef\;\left(i\right)\;X\;\left(i\right)$ (Coef stands for regression coefficients, which can also be represented by β, where β<0 is a protective variable, and β>0 is a risk factor. X represents the expression level of this dephosphorylated gene in glioma patients).

The risk score for each glioma patient was calculated using the previously described mathematical formula. Based on the median risk score, all glioma patients within the model were categorized into high-risk and low-risk groups. Kaplan-Meier (K-M) survival analysis was performed using the “survival” package in R and the “survminer” package to compare the Overall Survival (OS) differences between the two risk subgroups. The validity of the risk model was assessed by plotting the Receiver Operating Characteristic (ROC) curve using the “timeROC” package in R and calculating the Area Under Curve (AUC) value. The closer the AUC value is to 1, the more accurate the prediction model is.

### 2.3 Genetic variations in 12 selected genes in glioma

Mutation data of the DRGs selected for inclusion in the glioma prognostic risk model were queried online using the cBioPortal database. Using the R a waterfall plot of mutation information was produced, and the frequency and frequency of mutation occurrence were counted sequentially by mutation type and mutated gene, which was used to examine the mutation distribution of DRGs included in the risk model in gliomas. Meanwhile, heat maps were drawn to analyze the differences in expression of 12 DRGs in high and low risk groups.

### 2.4 Independent prognostic value of risk score model

According to the clinicopathological features, patients were divided into subgroups including age, gender, grade, new-event, cancer-status. The Chi-square test was applied to calculate the association between risk scores and clinicopathological parameters. In addition, univariate and multivariate Cox regression analyses were used to explore whether risk scores were independent prognostic indicators for glioma patients.

### 2.5 Construction and evaluation of a nomogram

In our study, clinicopathological factors such as risk score, age, gender, grade, new-event and cancer status were incorporated into the prognostic risk model. Column line plots were developed using the “rms”, “survival”, “survivor”, “surviminer”, and “timeROC” software packages were used to create line plots to predict the OS of glioma patients at 1, 3, and 5 years. In addition, ROC curves were plotted in this study to assess the excellence of the risk score among the many predictors. Calibration curves were plotted using R software for assessing the closeness between actual and predicted survival.

### 2.6 Gene set enrichment analysis

In order to explore the potential molecular mechanisms underlying the different prognoses of glioma patients in the high-risk and low-risk groups, GSEA was conducted separately to gain a deeper understanding of the differences in biological processes (BP), cellular components (CC), and molecular functions (MF) involved in gene sets between the two groups of patients. Gene set files were downloaded from the GSEA Database (v4.3.3), including 7608 gene sets for BP, 1026 gene sets for CC and 1820 gene sets for MF. After 1000 repetitions of the analysis, data with NOM *P* value<0.05 and FDR<0.25 were considered informative. The top five most active biological processes or signaling pathways were selected for presentation in this study.

### 2.7 Analysis of immune cell infiltration and immunotherapy

The tumor microenvironment (TME) is the microenvironment surrounding a tumor and consists of a protective microecosystem of tumor cells and surrounding stromal cells. To gain insight into the differences in immune cell infiltration between the high and low risk groups, three different deconvolution algorithms (EPIC, QUANTISEQ, and CIBERSORT) were used in this study to analyze the glioma cohort samples for immune cell infiltration, and the results of the analyses are represented by violin plots.

In addition, the ESTIMATE algorithm was used to predict the Immune Score and Stromal Score for each sample. In our study, we calculated the Tumor Mutation Burden (TMB) of glioma patients in the high- and low-risk groups based on somatic mutation data, and assessed whether there was a difference in TMB scores between the two groups of glioma patients. Tumor Immune Dysfunction and Exclusion (TIDE) scores were used to assess the ability of patients in the high- and low-risk groups to escape from tumor immunity.

### 2.8 Chemotherapy drug sensitivity analysis

Potential chemotherapeutic agents for the treatment of glioma were screened from Genomics of Drug Sensitivity in Cancer (GDSC) (https://www.cancerrxgene.org/). Bortezomib, Erlotinib, Etoposide, Imatinib, Tamoxifen, Temozolomide, and Vincristine were selected as candidate chemotherapeutic agents. The half maximal inhibitory concentration (IC_50_) of the chemotherapeutic drugs was calculated using the pRRophetic package in R. The IC_50_ was estimated by ridge regression with all parameters set at default values, using the batch effect of COMBAT and the tissue type of ALL, and duplicate gene expression was summarized as the mean value. Subsequent correlation analysis was performed between the 7 chemotherapeutic candidates and the IC_50_ values of the 12 DRGs for which risk score models were constructed.

### 2.9 Cell culture

The human U251 and LN229 cell lines with STR identification certificates used in this experiment were purchased from Sichuan Yuankangsheng Technology Co., Ltd. CDC25A overexpression plasmid and siRNA purchased from Linmei Biotechnology Co., Ltd. The complete medium used for cell culture was prepared by mixing 10% fetal bovine serum with 1% double antibody (penicillin-streptomycin mixture). The specific parameters of the cell culture incubator were 5% CO_2_ and kept constant, 37°C, and constant humidity. Passaging operations were performed approximately every three days depending on the density under the microscope, the current state, and the needs of the experiment.

### 2.10 Quantitative Real-Time PCR detection of mRNA expression of 12 DRGs

RNA was extracted from human brain normal glial cells (HEB) and glioma U251, LN229 cell lines using Foregene mRNA kit, and reverse transcription was performed with TaKaRa Reverse Transcription Kit. Real-time qPCR assay for the mRNA expression of 12 DRGs in HEB, U251 and LN229 cell lines. According to the SYBR Green qPCR Master Mix instructions, the two-step method was chosen to analyze the results using comparative Ct values with GAPDH as an internal reference. Histograms were plotted using GraphPad Prism 8 software, and t-tests were performed to analyze the results for significance. The primers for the 12 DRGs are shown in **Table 1**, in which the primer for CDC25A was quoted from Liang Huang et al. The primers for the remaining 11 genes were designed in the Sangon Biotech (https://store.sangon.com/).

**Table 1:** Primer Sequences.

Gene	FORWARD	REVERSE
LRRK2	CGGATGTTGGTGATGGAGTTAGC	GTTCTAGTGAGGCTGGCTTTGTC
PLPPR3	ATCGAATGGGACCCACCTGT	TTTGGATGGACTCGGAGGCA
MTMR11	ACAGCGGAAGACTGGGAGACTG	TGGTGGCTACGTCGAACCTCTC
LPIN3	CGCCTCTCCTCCGATCAGATCC	TGCCCTGGTACTGAGTGGTCAC
HDDC2	TGTGAAGCAGCTAGACCAATGTG	GCAGTCTCCCAGGTTTGTGTTC
GNA12	TCAGAGGCTGCTTGTGGTCT	AAGAGCCCTTGTGTGCTACTG
DUSP4	GCTGATGAACCGGGACGAGAATG	CAGTCCAGCAGCAGGCACTTG
DUSP21	CAACAACGGCTTTTGGGAACAGC	CTACCGGCGAGTTGATCATGCG
CDCA2	GCTGACTGTGTAGTGGGCAAAGG	TGGTGACCTGACATCAGGGACTG
CDC25B	GGAACGAGACCGTGCTGTCAAC	TCGGGTGCTGAGGGAAGAACTC
BMP2	TGACGAGGTCCTGAGCGAGTTC	ACCACGGCGTCCCTGCTG
CDC25A	TTCCTCTTTTTACACCCCAGTCA	TCGGTTGTCAAGGTTTGTAGTTC
GAPDH	TGTGTCCGTCGTGGATCTGA	CCTGCTTCACCACCTTCTTGA

### 2.11 qRT-PCR and western blot validation of transfection models

U251 and LN229 cells were transfected according to the instructions of Lipo3000 transfection reagent, and overexpression and knockdown cell models were constructed. The method of qRT-PCR was used to analyze whether the overexpression and knockdown cell models were constructed successfully. In addition, protein samples were extracted using RIPA lysis buffer, SDS-PAGE gels were prepared, samples were uploaded for electrophoretic separation, and then treated with CDC25A antibody or b-actin antibody at 4°C temperature overnight. The next day, the samples were exposed to a matching dilution of the secondary antibody and incubated at room temperature for 2h. Subsequently, the samples were developed using the ECL kit, and the protein expression levels were analyzed using ImageJ software.

### 2.12 Measurement of IC_50_

U251 and LN229 cells in logarithmic growth phase were inoculated into 96-well plates at 5×10^3^ cells per well and transfected. After 48h, TMZ was dissolved in dimethyl sulfoxide and diluted using complete medium to formulate it into a concentration gradient of 0, 0.5, 1, 2, 4, 8, 16, 32, 64, and 128 μg/mL. When the glioma cells grew to a density of about 70%, 100μL of configured TMZ was added to the cells according to the concentration gradient, and three replicate wells were set up for each concentration and a blank group was set up as the background. After continuing the incubation for 48h, 10μL of CCK-8 reagent was added to continue the incubation for 1h. Based on the effects of different drug concentrations on cell growth, the cell viability graphs were plotted and the IC_50_ values of the two cell lines were calculated separately.

### 2.13 Statistical analysis

All data processing, statistical analysis, and plotting in this experiment were performed using R software version 4.2.2 (version 4.2.2, https://www.r-project.org/). Experimental data were drawn and analyzed by GraphPad (Prism 8). T-test is used to compare whether there is a significant difference between the mean values of the two groups of data, which is expressed as mean ± standard deviation SD (*P<0.05, **P<0.01, ***P<0.001, ****P<0.0001, Ns, there was no statistical difference).

## 3 Results

### 3.1 Screen of DRGs in patients with glioma

**Figure 1:**
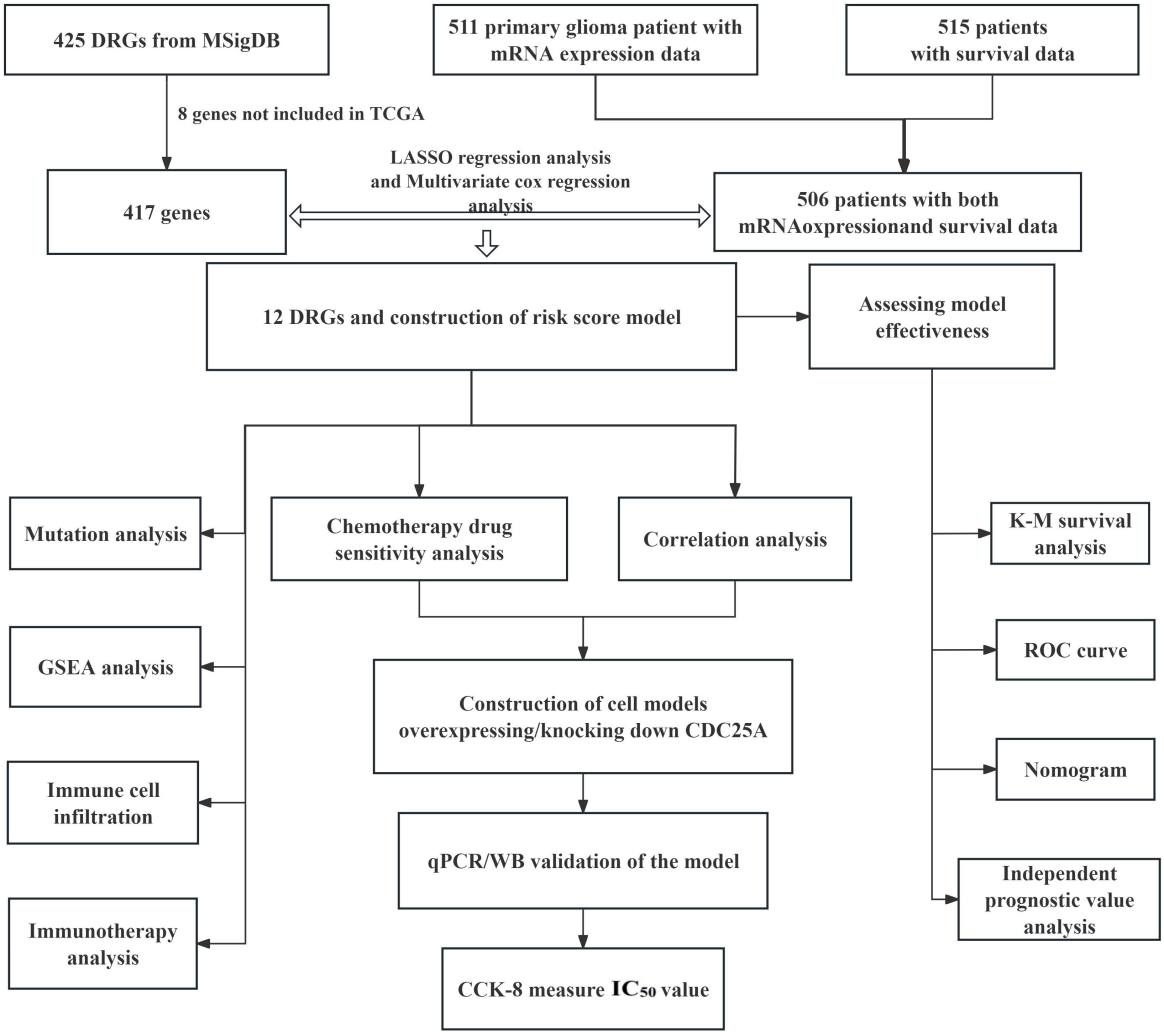
Flow chart of this study.

**Figure 2:**
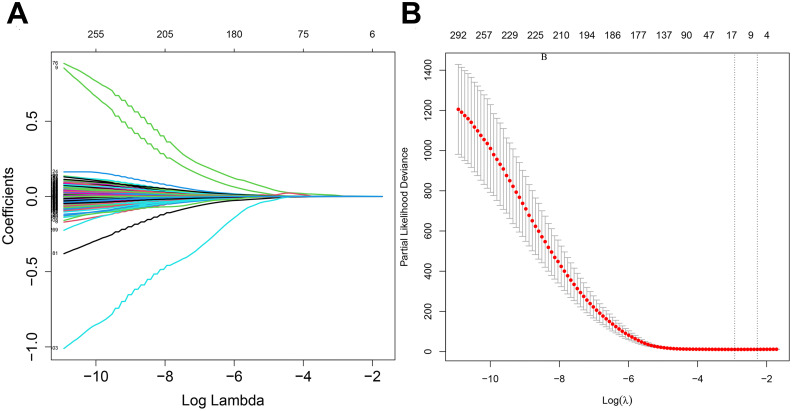
**(A)** LASSO regression modelling of cvfit. **(B)** LASSO regression modelling of lambda functions.

The flow chart of this study is shown in **Figure 1**. All the genes expressed by glioma patients after standardized processing were combined with the expression data of the latest released transcriptome of glioma patients in the TCGA database and the clinical data of the corresponding patients. LASSO regression analysis (**Figures 2A, B**) and multifactorial COX regression analysis were used to conduct an in-depth study of DRGs and 12 genes were successfully identified as closely related to the prognosis of glioma patients genes that are closely related to the prognosis of glioma patients (BMP2, CDC25A, CDC25B, CDCA2, DUSP21, DUSP4, GNA12, HDDC2, LPIN3, LRRK2, MTMR11, PLPPR3) (**Table 2**).

**Table 2:** The 12 DRGs details in the prognostic risk model.

mRNA	Ensemble ID	Chromosome location	β(Cox)	HR(95%CI)	p
BMP2	ENSG00000125845	Chr20: 6,767,686-6,780,246	-0.283	0.75(0.64-0.89)	<0.001
CDC25A	ENSG00000164045	Chr3: 48,157,146-48,188,417	0.621	1.86(1.24-2.79)	<0.001
CDC25B	ENSG00000101224	Chr20: 3,786,772-3,806,121	0.390	1.48(0.97-2.24)	0.068
CDCA2	ENSG00000184661	Chr8: 25,459,199-25,507,911	-0.413	0.66(0.45-0.97)	0.036
DUSP21	ENSG00000189037	ChrX: 44,844,021-44,844,888	7.350	1556.45(16.29-148747.1)	0.002
DUSP4	ENSG00000120875	Chr8: 29,333,064-29,350,684	0.116	1.12(0.97-1.3)	0.116
GNA12	ENSG00000146535	Chr7: 2,728,105-2,844,308	0.329	1.39(1.01-1.91)	0.043
HDDC2	ENSG00000111906	Chr6: 125,219,962-125,302,078	-1.094	0.33(0.18-0.64)	<0.001
LPIN3	ENSG00000132793	Chr20: 41,340,821-41,360,582	0.263	1.3(1.05-1.62)	0.018
LRRK2	ENSG00000188906	Chr12: 40,196,744-40,369,285	0.221	1.25(1.04-1.5)	0.019
MTMR11	ENSG00000014914	Chr1: 149,928,651-149,936,879	0.219	1.24(0.97-1.6)	0.089
PLPPR3	ENSG00000129951	Chr19: 812,488-821,955	-0.177	0.84(0.72-0.97)	0.021

### 3.2 Establishment and validation of the 12 DRGs prognostic signature

**Figure 3:**
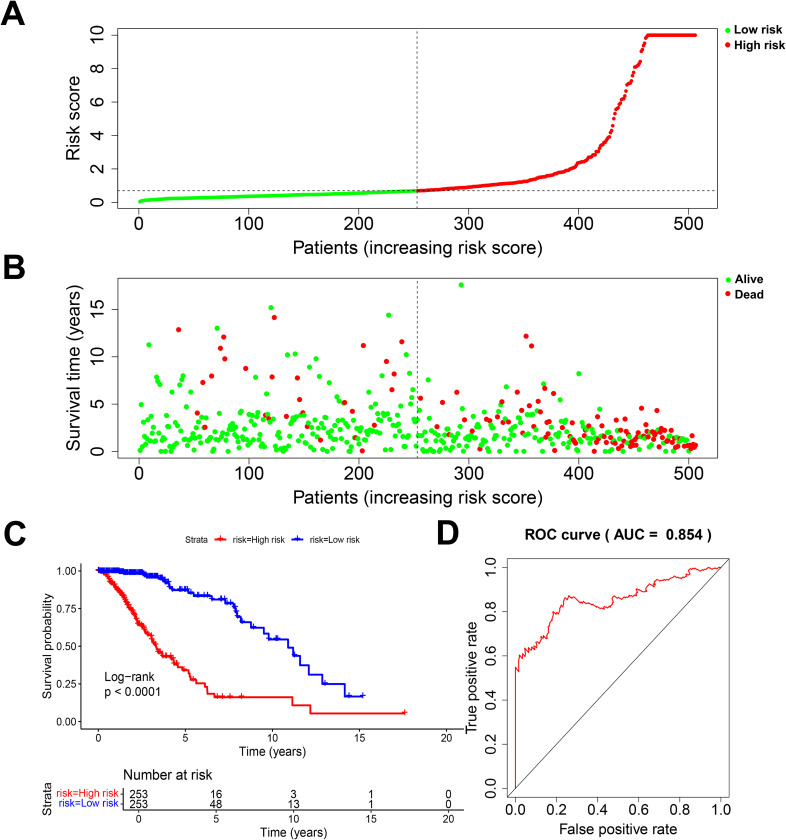
Evaluation of prognostic risk model. **(A)** Risk score plot. **(B)** Scatter plot of survival status. **(C)** K-M survival plot for patients in the high and low risk group. **(D)** 5-year diagnostic efficacy ROC plot.

According to the standardized gene expression values and their coefficients, a risk score was created. Risk score = (-0.2825×BMP2) + (0.6210×CDC25A) + (0.3898×CDC25B) + (-0.413292995×CDCA2) + (7.350161893×DUSP21) + (0.115914978×DUSP4) + (0.329334452×GNA12) + (-1.094422324×HDDC2) + (0.263233031×LPIN3) + (0.220752276×LRRK2) + (0.2185904×MTMR11) + (-0.177032997×PLPPR3). Using the above formula, the risk scores of all glioma patients were calculated, and the median risk value was selected to classify the patients into high-risk and low-risk subgroups (**Figure 3A**). The survival scatter plot of the risk scores showed that the number of deaths continued to increase with the gradual rise of the risk scores (**Figure 3B**). K-M survival analysis showed that patients in the low-risk group had a significantly higher OS than those in the high-risk group, and the prognosis was significantly different (**Figure 3C**, *P*<0.0001). The AUC value is the geometric area at the bottom right of the ROC curve, and the value range is 0 to 1, which means that the AUC is effective for testing the accuracy of the model. In general, an AUC of 0.5 indicates no difference, accuracy is low at 0.50-0.70, 0.7 to 0.8 is considered acceptable, 0.8 to 0.9 is considered excellent, and greater than 0.9 is considered excellent (29). By applying the ROC curve to assess the predictive ability of the risk score, we found that the AUC reached 0.854 over a 5-year period (**Figure 3D**). This indicates that the prognostic model constructed from 12 DRGs has high accuracy for prognosis prediction in glioma patients.

### 3.3 Genetic variation and gene expression of 12 DRGs

**Figure 4:**
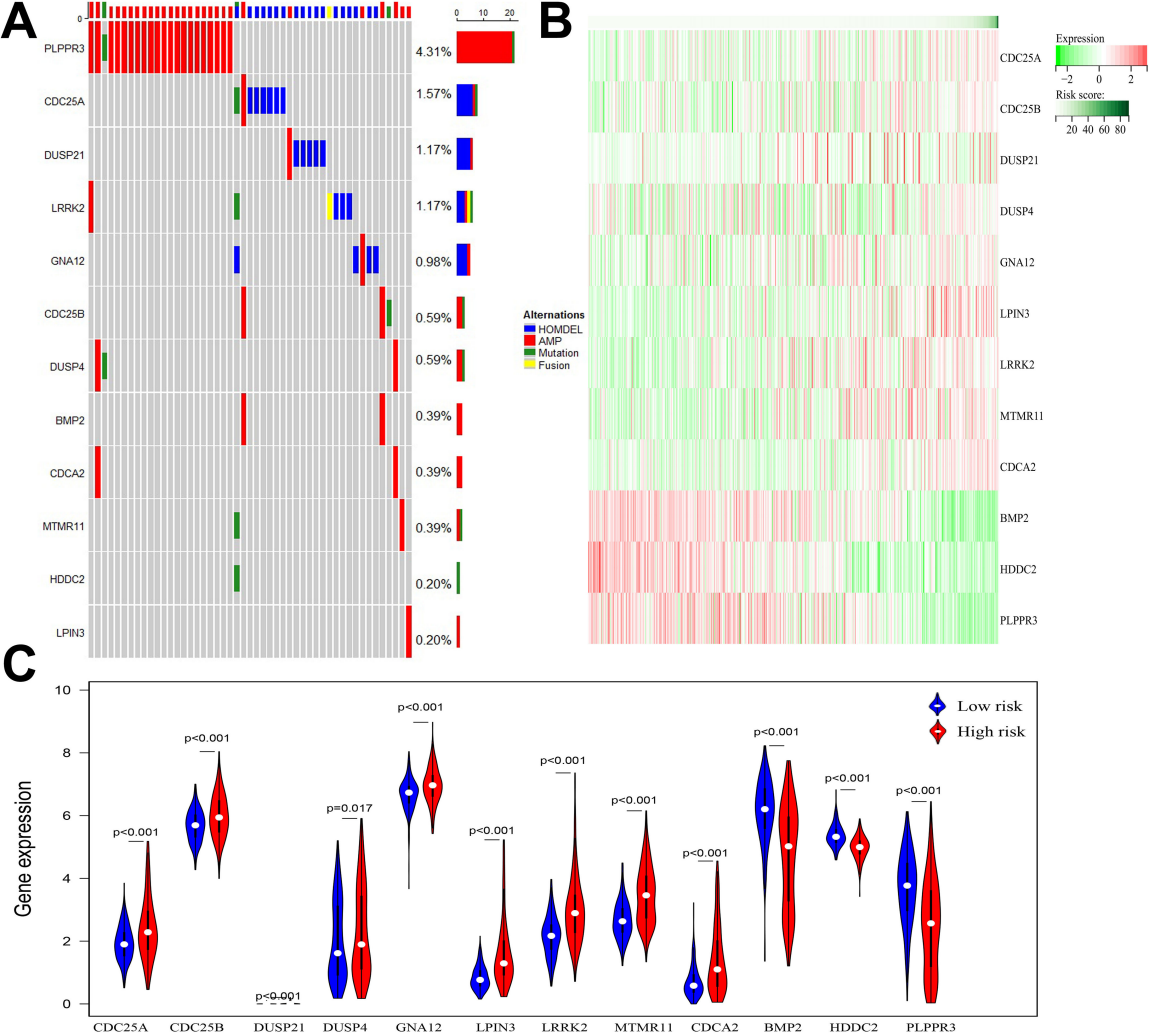
**(A)** Waterfall diagram displaying mutated gene names and mutation types in glioma patients. **(B)** Heatmap of 12 DRGs expressed in high and low risk groups. **(C)** Violin plot of 12 DRGs expressed in high and low risk groups.

Among the 511 patients included in this study, 49 (9.59%) of all queried genes altered. Of these, 28 cases (5.48%) had amplification, 17 cases (3.33%) had homdel, 2 cases (0.39%) had mutation, 1 cases (0.20%) had fusion, and 1 (0.20%) had multiple alterations, with amplification being the most common type of mutation among them (**Table 3**). Simultaneously, the waterfall plot showed that PLPPR3 had the highest mutation rate of 4.31%. Among the 511 glioma patients, 21 patients developed amplification, and 1 patient developed fusion. The mutation rate of CDC25A gene was 1.57%. Among 511 glioma patients, 6 had HOMDEL, 1 had Amplification and 1 had Fusion (**Figure 4A**). Details of the specific mutations in the 12 DRGs are shown in **Table 4**. The heat map results show that these 12 genes in the two groups have a remarkably differences in expression. The expression of CDC25A, CDC25B, CDCA2, DUSP21, DUSP4, GNA12, LPIN3, LRRK2, MTMR11 in low-risk group was lower than that in hieg-risk group. The expression of BMP2, HDDC2 and PLPPR3 in low-risk group was higher than that in high-risk group (**Figures 4B, C**).

**Table 3:** Alterations of 12 query DRGs in detailed mutation type.

Alteration	Number of casea	Frequency
Amplification	28	5.48%
HOMDEL	17	3.33%
Mutation	2	0.39%
Fusion	1	0.20%
Multiple alterations	1	0.20%
Total	49	9.59%

**Table 4:** Details of mutations in 12 DRGs in glioma patients (n=511).

mRNAs	No Alterations	Amplification	HOMDEL	Mutation	Fusion	Altered/Profiled(%)
BMP2	509	2	0	0	0	0.39%
CDC25A	503	1	6	0	1	1.57%
CDC25B	508	2	0	0	1	0.59%
CDCA2	509	2	0	0	0	0.39%
DUSP21	505	1	5	0	0	1.17%
DUSP4	508	2	0	0	1	0.59%
GNA12	506	1	4	0	0	0.98%
HDDC2	510	0	0	0	1	0.20%
LPIN3	510	1	0	0	0	0.20%
LRRK2	505	1	3	1	1	1.17%
MTMR11	509	1	0	0	1	0.39%
PLPPR3	489	21	0	0	1	4.31%

### 3.4 Independent prognostic value of risk scoring models

**Figure 5:**
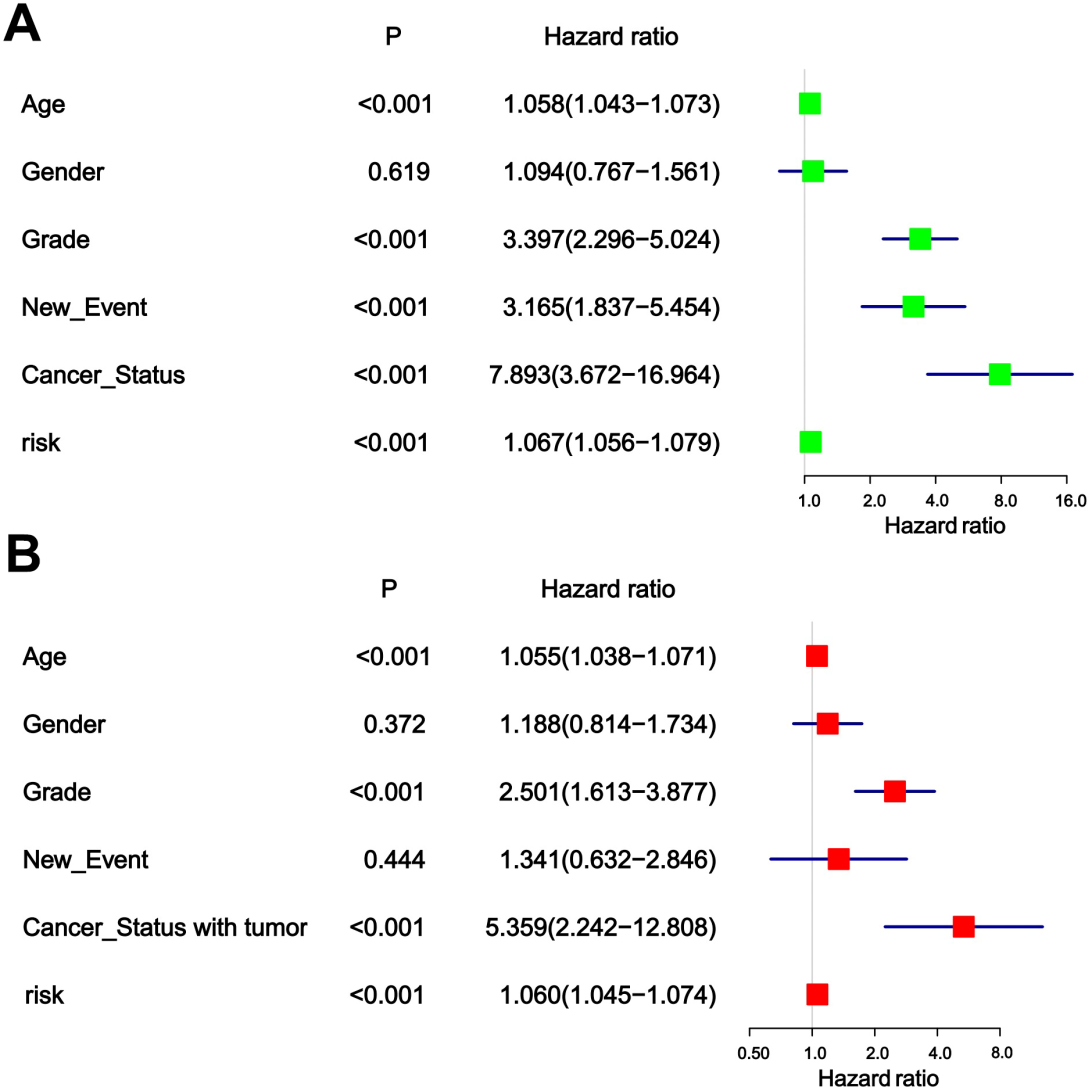
**(A)** Univariable analyses for clinical feature and risk score. **(B)** Multivariable analyses for clinical feature and risk score.

The chi-square test found that age, tumor grade, tumor status, and new-event were significantly associated with risk scores in patients with glioma (**Table 5**). Among them, the results of univariate COX regression analysis suggested that patient’s age, grade, new event and tumor status were significantly associated with OS in glioma patients (*P*<0.001) (**Figure 5A**). Subsequently, multifactorial COX regression analysis further revealed that risk score (*P*<0.001), age (*P*<0.001), tumor grade (*P*<0.001), cancer status with tumor (*P*<0.001) were independent risk factors for OS in glioma patients and all of them were positively associated with the risk of death in glioma patients (**Figure 5B**).

**Table 5:** The relation between risk score and clinical features.

Variables	Total(n=506)	Highrisk(n=253)	Lowrisk(n=253)	*p*
Age, Median (Q1,Q3)	41(32.25,53)	43(33,57)	39(32,50)	0.01
Gender, n (%)				0.245
FEMALE	226(45)	120(47)	106(42)	
MALE	280(55)	133(53)	147(58)	
Grade, n (%)				<0.001
G2	245(49)	88(35)	157(62)	
G3	260(51)	164(65)	96(38)	
New_Event, n (%)				<0.001
NO	231(46)	96(38)	135(53)	
YES	275(54)	157(62)	118(47)	
Cancer_Status, n (%)				0.002
Tumor free	175(39)	72(32)	103(47)	
With tumor	271(61)	154(68)	117(53)	

### 3.5 Construction and correction of nomogram

**Figure 6:**
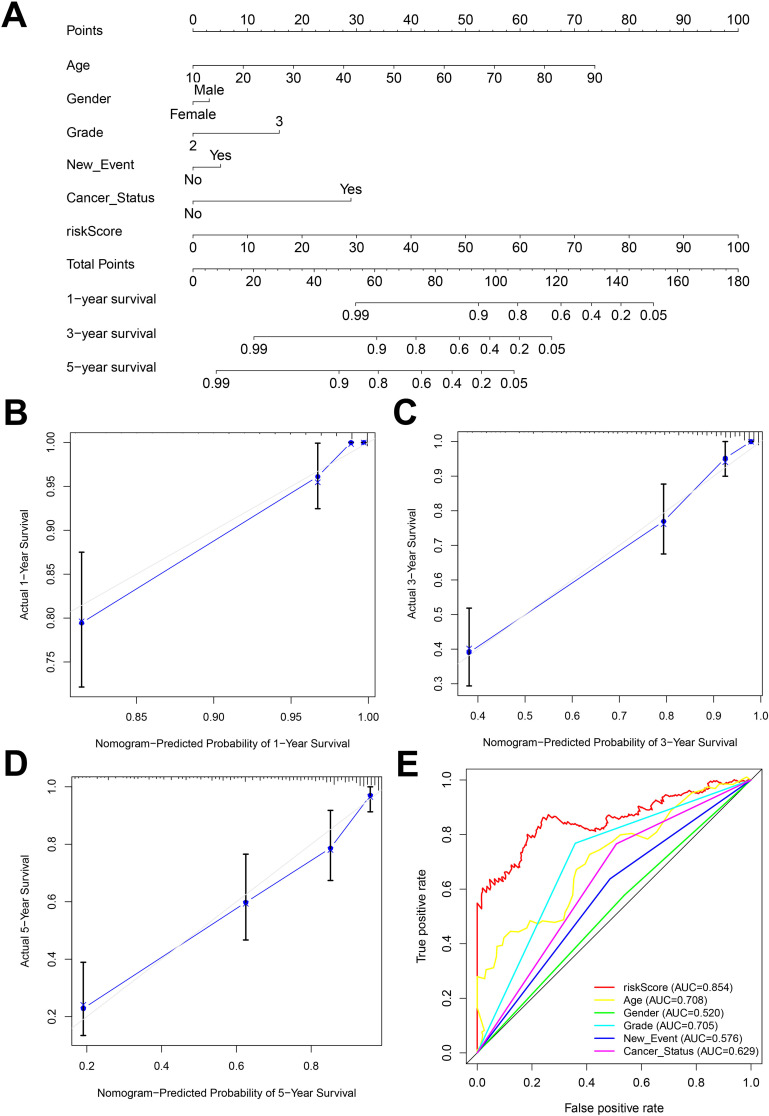
**(A)** Nomogram constructed by combining risk score and clinicopathologic characteristics. **(B-D)** Standard versus calibrated graphs for predicting 1-, 3-, and 5-year OS in glioma patients. **(E)** Standard ROC plots of risk scores and clinicopathologic features.

Nomogram is an important tool for the combined diagnosis or prediction of the onset or progression of a disease by multiple indicators. In our research, nomogram was constructed to predict the OS of glioma patients at 1, 3 and 5 years based on age, gender, grade, new-event, cancer status and risk score (**Figure 6A**). The calibration curves for the 1-, 3- and 5-year forecasts showed a high degree of fit to the standard curve, indicating a high degree of consistency between the forecasts and the actual results (**Figures 6B–D**). In addition, the AUC values for the risk score, age, gender, grade, new event, tumor status established in this study were 0.854, 0.708, 0.520, 0.705, 0.576, 0.629, respectively, indicating that the AUC values for the risk score were higher than the other predictors and the predictive effect was more excellent (**Figure 6E**).

### 3.6 Gene enrichment analysis results

**Figure 7:**
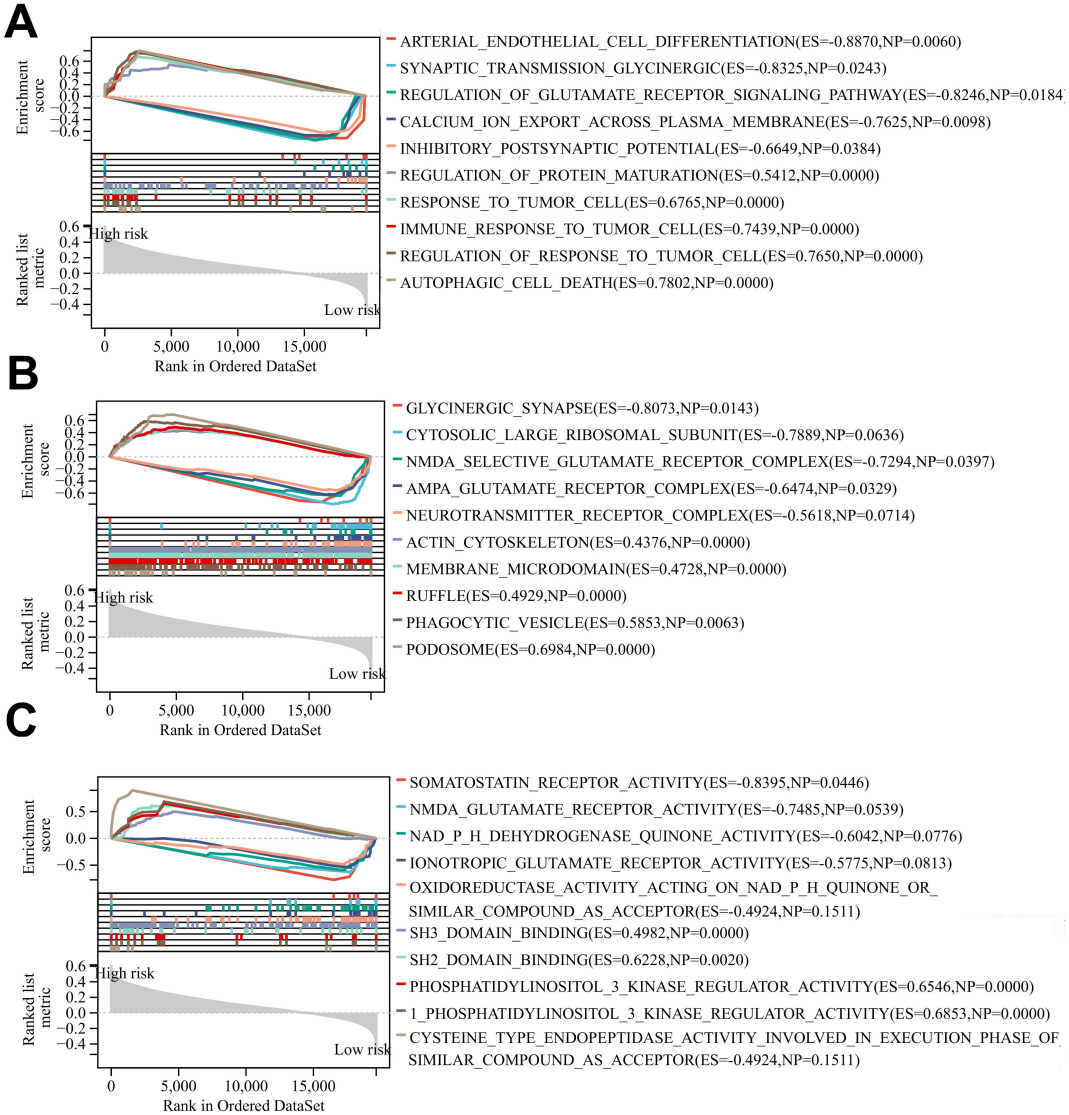
**(A-C)** BP, CC and MF enrichment analysis of glioma patients in the high-risk group and low risk group.

The results of BP, CC, and MF analyses showed that the high-risk group was more active in biological processes such as regulation of glutamate receptor signaling pathways, inhibitory postsynaptic potential, actin cytoskeleton, phagocytic vesicles, cysteine-type endopeptidase activity in the apoptotic execution phase, and binding of SH3 structural domains (**Figures 7A–C**), and the low-risk group was enriched in biological processes such as modulation of cellular receptor signaling pathways, positive modulation of the organization of extracellular matrix, negative modulation of immune effector processes negative regulation, glutamate receptor complex, glycinergic synapses, NADPH dehydrogenase quinone activity, oxidoreductase activity activation of paired donors, and other biological processes were enriched (**Figures 7A–C**). The above differences in the results of GSEA analysis between the two groups may reveal the intrinsic reasons leading to the differences in the prognosis of glioma patients in the high- and low-risk groups, and the specific mechanisms need to be further investigated.

### 3.7 The landscape of tumor microenvironment immune cell infifiltration in the two risk groups

**Figure 8:**
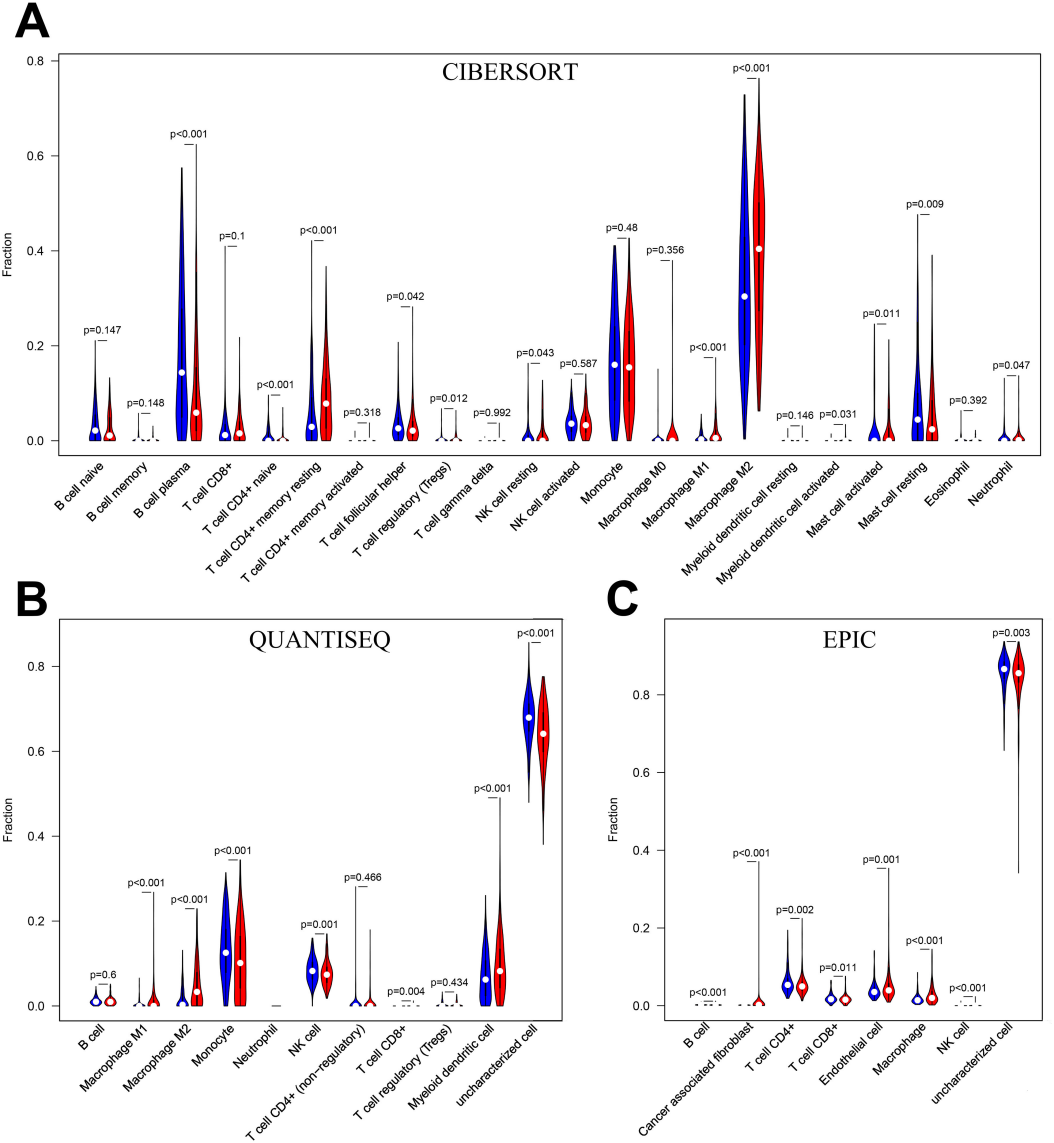
**(A)** Immune cell infiltration analysis in CIBERSORT. **(B)** Immune cell infiltration analysis in QUANTISEQ. **(C)** Immune cell infiltration analysis in EPIC. Blue for low-risk group, red for high-risk group.

B cell plasma, T cell CD4+ naïve, T cell follicular helper, mast cell resting and neutrophils infiltrated significantly more in the low-risk group than in the high-risk group in the CIBERSORT algorithm (*P*<0.05) (**Figure 8A**). monocyte, NK cell and uncharacterized cell infiltrated significantly more in the low-risk group than in the high-risk group in the QUANTISEQ algorithm (**Figure 8B**). B cell, T cell CD4+, T cellCD8+ and uncharacterized cell in the EPIC algorithm were infiltrated higher in the low-risk group than in the high-risk group (**Figure 8C**). The above results suggest that there is a difference in the level of immune cell infiltration in glioma patients in the high- and low-risk groups, which in turn affects the tumor immune microenvironment.

### 3.8 Immunotherapy analysis results

**Figure 9:**
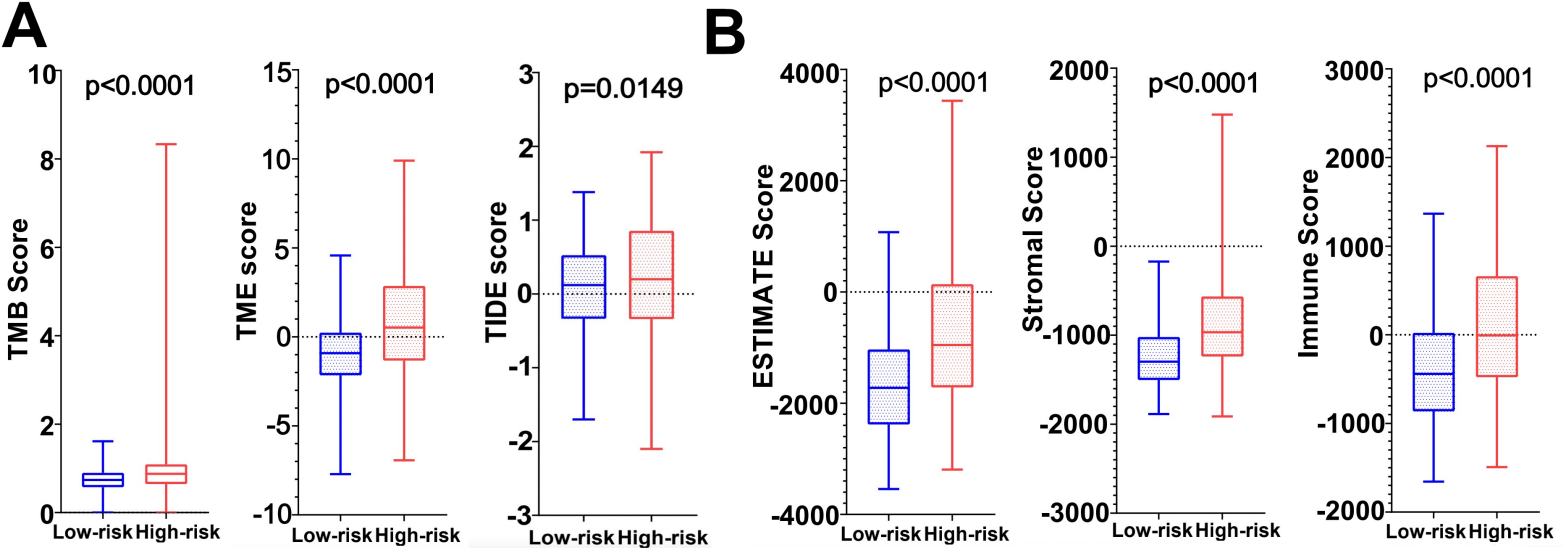
**(A)** TMB score, TME score and TIDE score in high and low risk groups. **(B)** Tumor microenvironment scores (ESTIMATE Score, Stromal Score and Immunity Score) in the high and low risk groups.

Immunotherapy correlation analysis showed that the TIDE Score, TME Score, and TMB Score of glioma patients in the high-risk group were significantly higher than those in the low-risk group (*P*<0.05) suggesting that the high-risk patient group possessed higher immune evasion and relatively poorer immunotherapy (**Figure 9A**). The Immune Score, Stromal Score and ESTIMATE Score were all significantly higher (*P*<0.0001) than those of the low-risk group, revealing a significant difference in TME between the two groups, suggesting that the high-risk group had a higher percentage of immune and stromal cells (**Figure 9B**).

### 3.9 qRT-PCR results of 12 DRGs in 3 groups of cells

**Figure 10:**
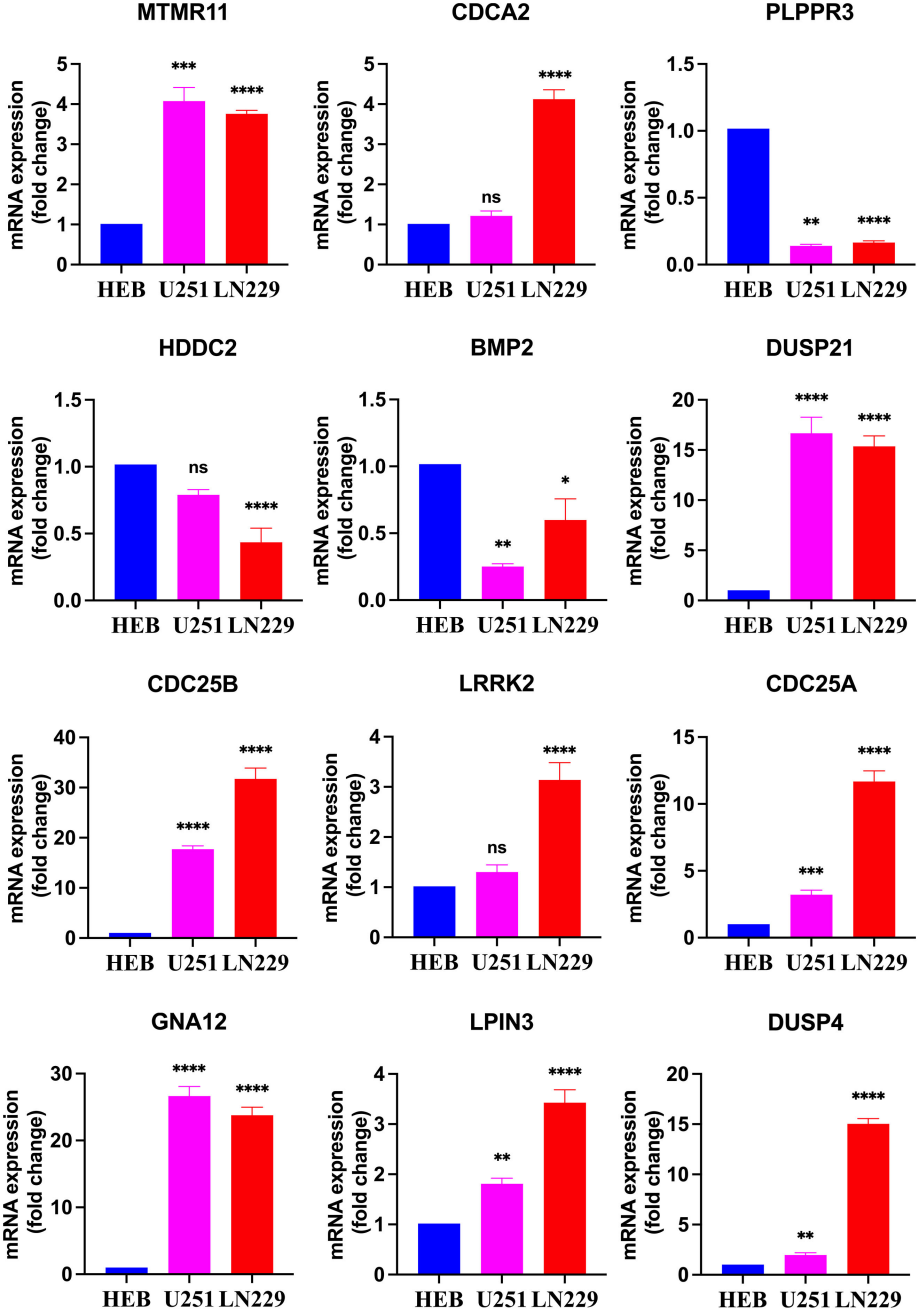
Real-time qPCR detection of mRNA expression of 12 DRGs in the HEB, U251 and LN229. *P<0.05, **P<0.01, ***P<0.001, ****P<0.0001, Ns, there was no statistical difference.

The results revealed that CDC25A, CDC25B, DUSP21, DUSP4, GNA12, LPIN3 and MTMR11 mRNA expression levels in U251 glioma cancer cells were significantly higher than those in HEB cells, and that BMP2, PLPPR3 mRNA expression levels were significantly lower than those in HEB cells. LN229 cells showed significantly higher expression levels of CDC25A, CDC25B, CDCA2, DUSP21, DUSP4, GNA12, LPIN3, LRRK2 and MTMR11 mRNA expression levels were significantly higher than those in HEB cells, BMP2, HDDC2 and PLPPR3 mRNA expression levels were significantly lower than those in HEB (**Figure 10**).

### 3.10 Drug sensitivity analysis and correlation results

**Figure 11:**
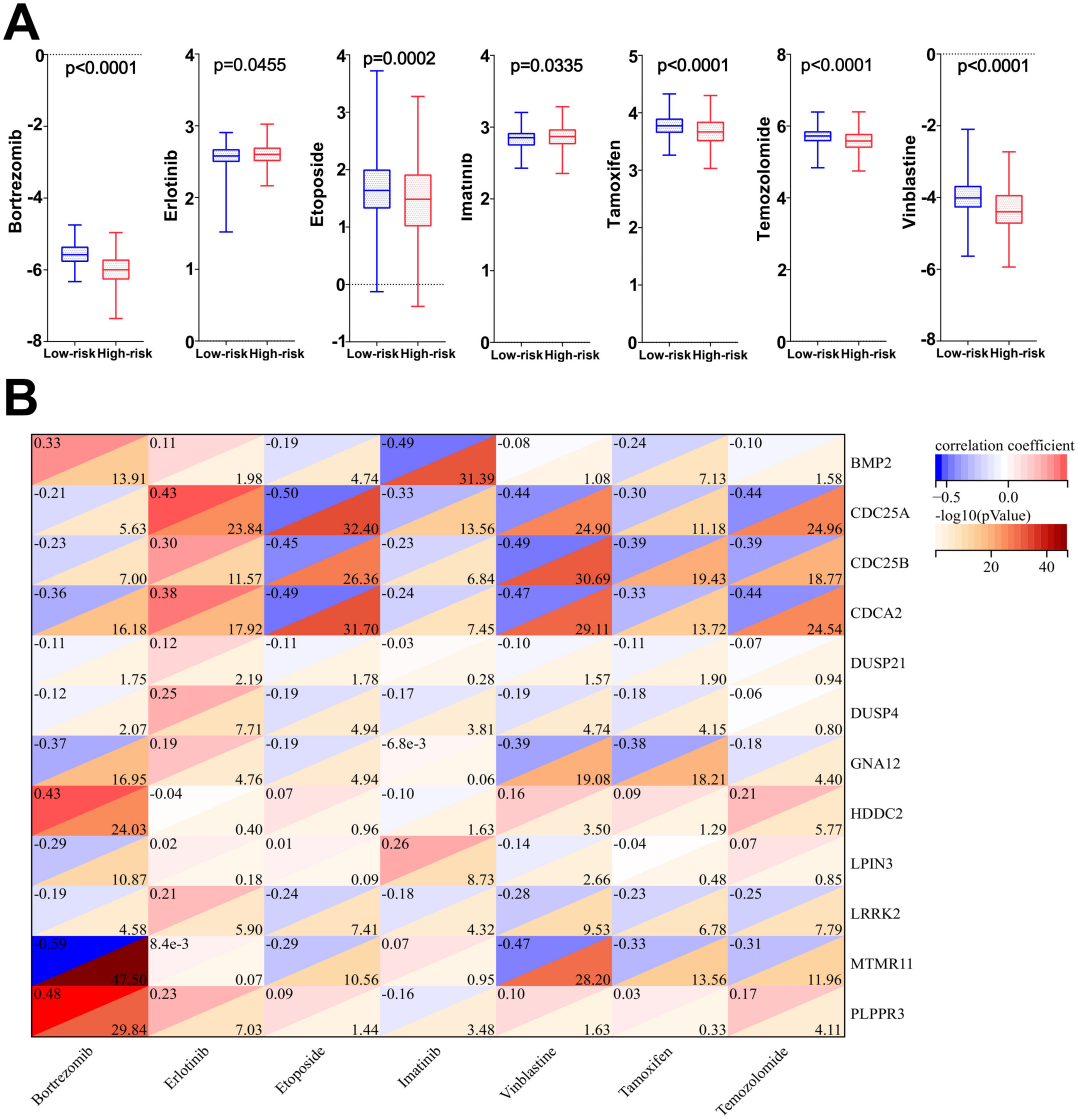
**(A)** Differences in IC_50_ values among the seven chemotherapeutic agents in the high- and low-risk groups. **(B)** Correlation analysis of 7 chemotherapeutic agents and 12 dephosphorylation-related genes.

The results of drug sensitivity analysis showed that the IC_50_ values of bortezomib, etoposide, tamoxifen, TMZ and vincristine were significantly lower in the high-risk scoring group compared with the low-risk scoring group (*P*<0.05) (**Figure 11A**), indicating that glioma patients in the high-risk group were more sensitive to bortezomib, etoposide, tamoxifen, TMZ and vincristine. Therefore, the model based on DRGs may have potential predictive value for the sensitivity to chemotherapeutic agents, providing assistance for clinical individualized treatment of glioma patients. In addition, this study performed a correlation analysis of the 7 chemotherapeutic agents commonly used in the clinical treatment of glioma and the 12 DRGs included in the risk model. The analysis showed that the IC_50_ value of the commonly used chemotherapeutic drug TMZ had the highest and negative correlation with the gene CDC25A (correlation: -0.44, P=-log10^24.96^) (**Figure 11B**). In addition, qPCR results has confirmed that the gene CDC25A was significantly higher expressed in glioma cells U251 and LN229 than in HEB cells (*P*<0.001, *P*<0.0001). Therefore, the genes CDC25A and TMZ were selected in this study for subsequent experimental validation.

### 3.11 Models of cells overexpressing and knocking down CDC25A

**Figure 12:**
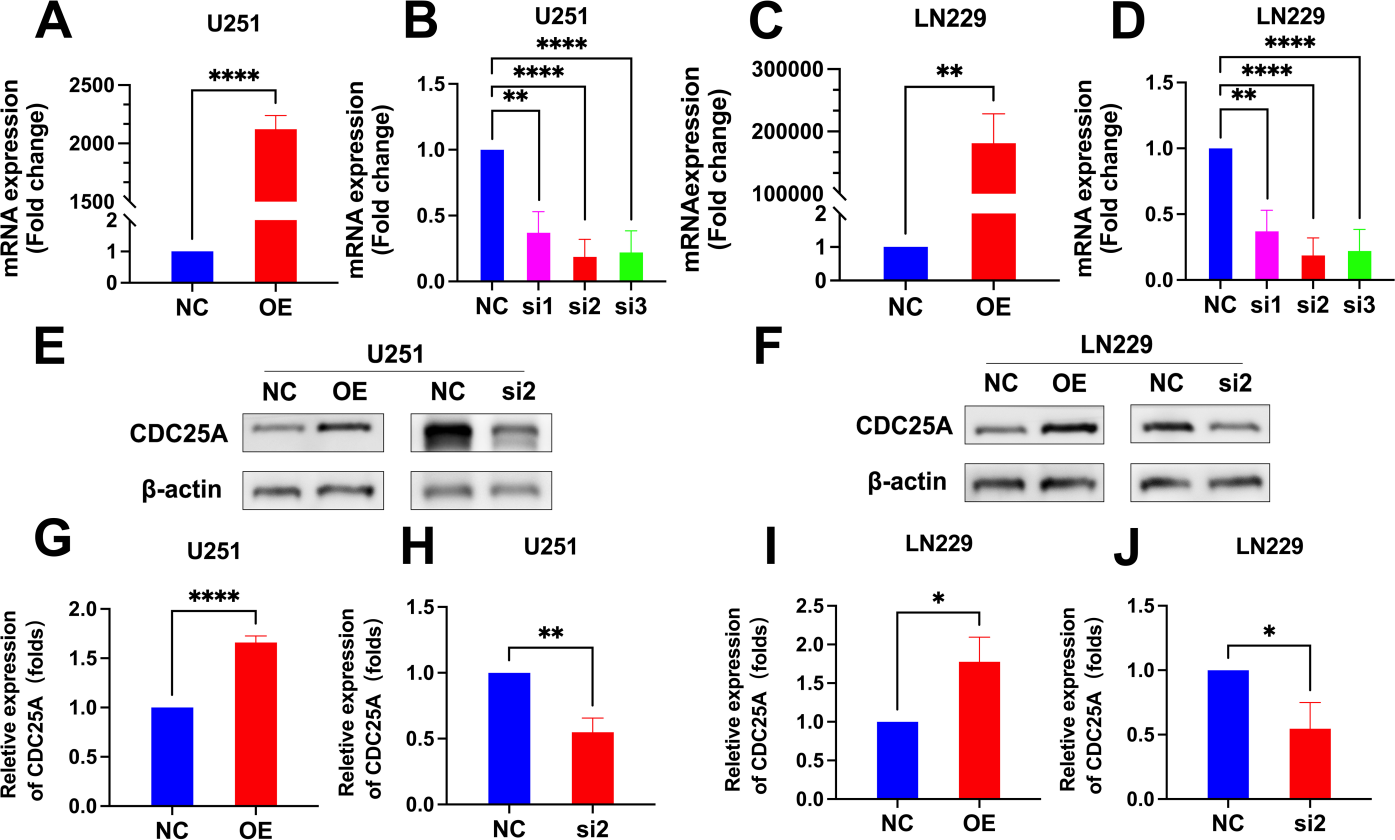
**(A-D)** qRT-PCR detection of CDC25A expression in U251 and LN229 cell line after overexpression/knockdown transfection. **(E-F)** Validation of overexpression and knockdown of CDC25A protein expression in U251/LN229 cell line. **(G-J)** Comparative histogram of proteins in the OE, NC, si2 and NC in the U251 and LN229 cell line. (*P<0.05, **P<0.01, ****P<0.0001.

The qRT-PCR results showed that the mRNA expression levels of CDC25A were significantly higher in the U251 and LN229 OE groups compared to the NC group (*P*<0.0001, *P*<0.01) (**Figures 12A, C**). mRNA expression levels of CDC25A were significantly lower in the U251 and LN229 knockdown groups, and the best knockdown effect was observed in the si2 group (*P*<0.0001, *P*<0.0001) (**Figures 12B, D**). Western Blot results are shown in the **Figures 11E, F**. The protein expression level of CDC25A in OE group of U251 and LN229 cells was significantly increased (*P*<0.0001, *P*<0.05) (**Figures 12G, I**). si2 protein expression level was significantly decreased in U251 and LN229 cells (*P*<0.01, *P*<0.05) (**Figures 12H, J**). The above results showed that U251 and LN229 cell models with overexpression and knockdown of CDC25A were successfully constructed. Subsequently, the cell model was used for subsequent TMZ drug sensitivity experiments. The results showed that overexpression of CDC25A reduced the resistance of U251 and LN229 to TMZ, and knockdown of CDC25A increased the resistance of U251 and LN229 to TMZ.

### 3.12 CDC25A affects the drug sensitivity of TMZ

**Figure 13:**
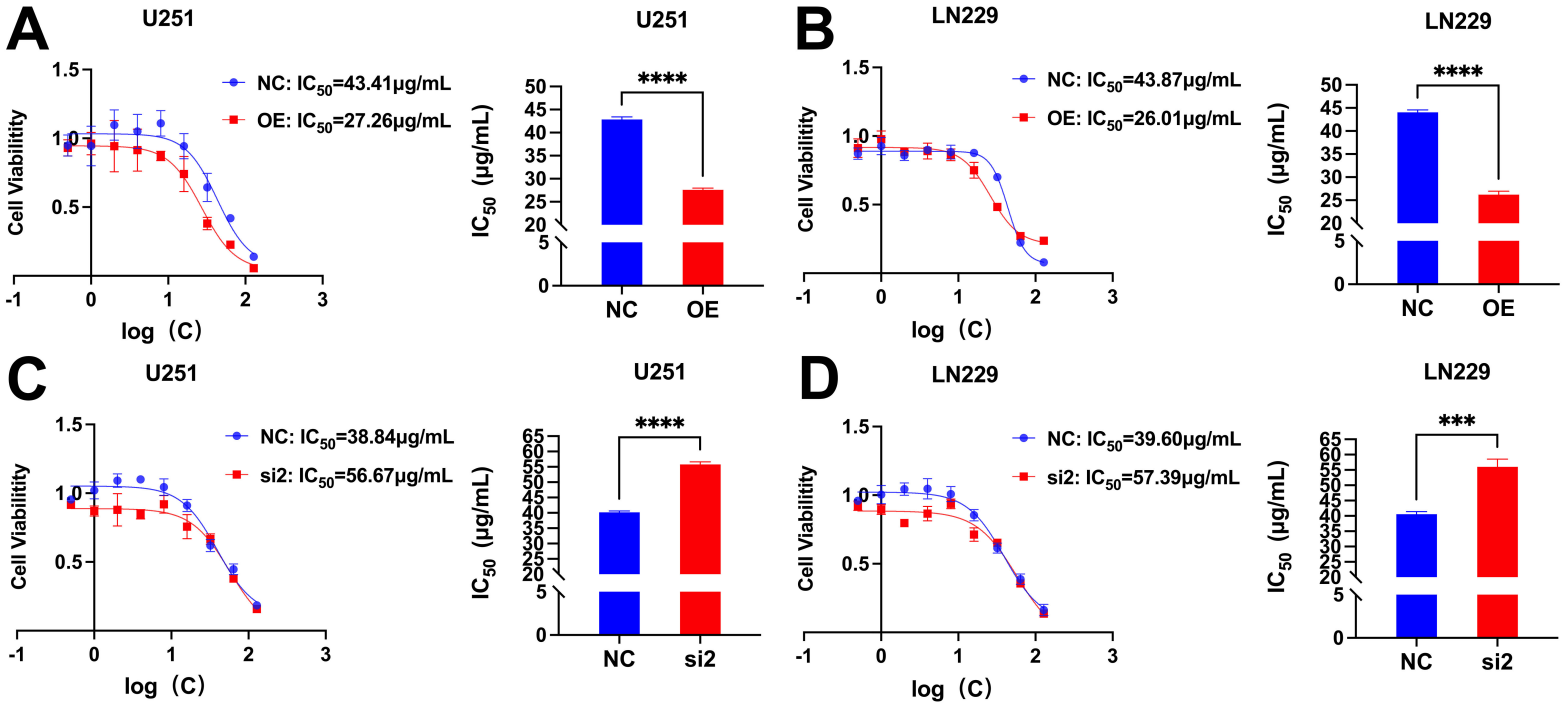
**(A-B)** Survival of U251/LN229 cell line in NC and OE groups after transfection. **(C-D)** Survival of U251/LN229 cell line in NC and si2 groups after transfection. ***P<0.001, ****P<0.0001.

The IC_50_ values of U251 and LN229 cells OE group were significantly decreased compared with NC group, suggesting that overexpression of CDC25A significantly increased the sensitivity of U251 and LN229 cell lines to TMZ (**Figures 13A, B**). In addition, the IC_50_ values of the U251 and LN229 cells si2 group were significantly increased compared with the NC group, suggesting that knockdown of CDC25A significantly decreased the TMZ sensitivity of the U251 and LN229 cell lines (**Figures 13C, D**).

## 4 Discussion

The strength of this research lies in being the first to establish a risk scoring model for glioma patients associated with DRGs which can precisely predict the prognosis of glioma patients. Additionally, in contrast to the existing glioma biomarkers (such as IDH mutation, 1p/19q code deletion, TERT promoter mutation, H3F3A mutation, etc.) and previous studies on glioma dephosphorylation, the novelty of this study is that it has been experimentally verified that CDC25A influences the TMZ resistance of glioma U251 and LN229 cells, potentially providing a reference for the clinical treatment of glioma patients with TMZ.

Among the 12 DRGs screened in this study CDC25A, CDC25B, CDCA2, DUSP11, DUSP4, GNA12, LPIN3, MTMR11, and LRRK2 were deleterious genes, whereas BMP2, HDDC2, and PLPPR3 were protective genes. Erna Raja et al. confirmed that BMP-2 is a putative tumor suppressor in Glioblastoma (GBM) (30). In addition, BMP2 was a protective factor in the prognostic models established in both studies by Xin Fan et al (31, 32). The above studies are consistent with the results of the present study. Phosphorylation level of CDC25A was correlated with malignancy and prognosis of gliomas in a study by Ji Liang et al (33). In a prognostic model developed by Melih Özbek et al. CDC25A was correlated with poor prognosis in patients with low-grade gliomas (34). YongJung et al. demonstrated that CDC25B can be used as a predictive biomarker for GBM (35). A pan-cancer analysis showed that high CDCA2 expression was associated with poor prognosis in low-grade gliomas (36). Two additional studies suggest that DUSP-4 may be a diagnostic and prognostic marker for IDH1 mutant gliomas, GNA12 signaling regulation promotes transcriptional and phenotypic responses to GBM tumor invasion (37, 38). Jing Yan et al. indicated that LRRK 2 increased the risk of low-grade gliomas, and that the lack of LRRK2 leads to impaired macrophage function and affects tumor progression in a cancer type-specific manner (39). The above related studies are consistent with the findings of the present study, further supporting the reliability of the prognostic model established in this study. Moreover, DUSP11, LPIN3, MTMR11, HDDC2, and PLPPR3 have not been queried for glioma-related studies. Therefore, they may become new biomarkers for the diagnosis and prognosis of glioma.

Presently, multiple studies have illustrated that the TME plays a pivotal role in the development of gliomas (40–42). The TME consists of tumor cells and surrounding stromal cells, which together form a protective microecosystem for its own proliferation, invasion, generation of therapeutic sensitivity, and immune escape (42–46). Despite significant clinical advances in tumor therapies, the survival rate of glioma patients has not been significantly improved, and the reason for this may be related to immune escape caused by TME (42, 44). The TME scores of the high-risk group were higher than those of the low-risk group and the percentage of immune cells and stromal cells was higher in the high-risk group in the results of the present study. The above may be potential factors contributing to the different prognoses of patients with high- and low-group gliomas. Furthermore, TMB is widely recognized as a predictive biomarker of immunotherapy efficacy. In this study, the TIDE score and TMB score of glioma patients in the high-risk group were significantly higher than those in the low-risk group, suggesting that the high-risk patient group had a stronger immune evasion potential and relatively poorer immunotherapy efficacy. In recent years, immune cells also play an important role in tumorigenesis and development. Neutrophils, an important component of TME, have been reported to be associated with malignant progression and immunosuppression in gliomas (47). Additionally, Macrophages have been shown to be actively involved in tumor growth and are the most extensively infiltrated immune cells in the TME (48). For example, Anna Gieryng’s study claimed that macrophages M2, play an immunosuppressive role in TME (49), and Hao Zhang et al. showed a negative correlation between survival and macrophages in glioma patients (50). The immune cell infiltration analysis in our study showed that both neutrophils and macrophages were infiltrated in the high-risk group. The above related studies further confirm the accuracy of the predictive model in this study. In addition, high infiltration of NK cells and CD4+ T cells, among others, in the low-risk group has also been reported for support. Chemotherapeutic drug sensitivity analysis showed that patients in the high-risk group were more sensitive to bortezomib, etoposide, tamoxifen, TMZ and vincristine demonstrating that the model developed in our study may have potential predictive value for chemotherapeutic drug sensitivity. Additionally, researchs on the gene CDC25A and chemotherapy drug resistance are gradually increasing. A study have shown that CDC25A promotes resistance to cisplatin and paclitaxel in ovarian cancer. Research by Liang Huang et al. revealed that Let-7 c-5p inhibits cisplatin resistance in lung adenocarcinoma cells by targeting CDC25A. Research by Yumi Ito et al. showed that inhibiting PCBP4 can reduce the resistance of human maxillary cancer cells to cisplatin (51–53). Our subsequent experiments confirmed that CDC25A may become a biomarker for evaluating TMZ resistance in gliomas, providing a new clinical treatment direction for drug-resistant glioma patients.

However, this study has some limitations and the established risk prognostic model has not been validated by an external database; in the future, it is hoped to be validated in a multicenter clinical trial. The experiments are relatively shallow and need to be further verified by more rigorous molecular biology experiments. In addition, previous cancer studies have suggested that the high expression of specific genes plays a key role in tumorigenesis. This view mostly underestimates the contribution of genes with lower expression levels. Such biases can have a profound impact on scientific understanding and clinical outcomes, as they can obscure the true complexity of cancer biology and limit the potential for discovering new therapeutic targets. The data extracted from the TCGA database in this study, a series of bioinformatics analysis and experimental verification results may also have such bias (54, 55). The association of high expression of CDCD25A with glioma does not necessarily imply a direct causal relationship and requires follow-up studies.

## 5 Conclusion

The DRGs risk model has a good prediction efficiency, which provides a valuable new direction for the prognosis prediction of glioma patients and the clinical use of TMZ chemotherapy drugs.

CDC25A affects TMZ resistance in glioma cells U251 and LN229. We will next continue to investigate the application of CDC25A in other glioma subtypes or therapeutic combinations.

## Data Availability

The original contributions presented in the study are included in the article/supplementary materials, further inquiries can be directed to the corresponding author/s.
